# Use of Ubp1 protease analog to produce recombinant human growth hormone in *Escherichia coli*

**DOI:** 10.1186/s12934-014-0113-4

**Published:** 2014-08-27

**Authors:** Anna Wojtowicz-Krawiec, Iwona Sokolowska, Maria Smorawinska, Luiza Chojnacka-Puchta, Diana Mikiewicz, Natalia Lukasiewicz, Alina Marciniak-Rusek, Renata Wolinowska, Anna Bierczynska-Krzysik, Anna Joanna Porebska, Jolanta Kuthan-Styczen, Lidia Gurba, Piotr Borowicz, Anna Mazurkiewicz, Grazyna Plucienniczak, Andrzej Plucienniczak

**Affiliations:** Institute of Biotechnology and Antibiotics, Staroscinska 5, Warsaw, 02-516 Poland; Department of Pharmaceutical Microbiology, Medical University of Warsaw, Oczki 3, Warsaw, 02-007 Poland

**Keywords:** Recombinant human growth hormone, Ubiquitin, Deubiquitinating protease, Protein expression

## Abstract

**Background:**

Numerous bacterial human growth hormone (hGH) expression methods under conventional fermentation and induction conditions have been described. Despite significant progress made in this area over the past several years, production of recombinant hGH by using cellular expression systems still requires further optimization. Fusion of the ubiquitin (Ub) tag to the hGH protein allowed to increase of the overall efficiency of the biosynthesis and improve the protein stability. Ub is a protein composed of 76 amino acid residues with a molecular mass of 8.6 kDa, expressed in all eukaryotes. This protein is an element of the universal protein modification system, which does not occur in bacteria, and is a useful carrier for heterologous proteins obtained through expression in *Escherichia coli.* Purification of Ub-fusion proteins is easier than that of unconjugated recombinant proteins, and Ub can be removed by deubiquitinating proteases (DUBs or UBPs).

**Results and Conclusion:**

In the present study the UBPD2C protease, a stable UBP1 analog, was produced as a recombinant protein in *E. coli* and used for production of recombinant human growth hormone (rhGH). hGH was expressed as a fusion protein with Ub as a tag. Our findings show that the UBPD2C protease is very effective in removing the Ub moiety from recombinant Ub-fused hGH. The described approach enables obtaining a considerable yield of rhGH in a purity required for pharmaceutical products.

## Background

UBP1 protease is a yeast cysteine protease, 809 amino acids long, which cleaves ubiquitin from proteins fused to its C-terminus [1], and binds to Ub through an ester bond during the reaction. The protease activity depends on its ability to cleave the Ub peptide fused via its C-terminus to other polypeptides, regardless of the amino acid sequence of the fused moiety [2].

In some expression systems, proteins of interest are first expressed as fusions with Ub [3] or its derivatives [4-10], and then recovered using an Ub-removing enzyme (e.g. UBP1) [11,12]. This approach has many advantages, including improved quality and efficiency of protein expression as well simplified purification process, which are of great importance in the industrial production of recombinant proteins [13-18]. We previously described [19] UBP1 protease deletion and point mutations, which improved the expression level of the protease in a microbiological expression system [20].

In technological processes, large quantities of deubiquitylating enzyme (DUB) are required for maximum possible catalytic activity. However, the majority of the currently available expression protocols does not lead to efficient expression of DUB, which greatly limits their applicability, especially in industrial processes. In the production of a specific protein for therapeutic application, particularly human growth hormone (hGH), purity and activity of the enzymes used are important factors. Accordingly, we have presented various solutions to meet these requirements [19,20]. Nevertheless, there is still a need for a method to obtain highly active DUB enzyme in a gram-scale quantity for the production of bioactive peptides [4,5,21].

In the present study, we describe the development of an efficient method for obtaining hGH from bacterial cells, particularly from *Escherichia coli*. This method could yield gram-scale quantities of the protein with appropriate activity for application in the production of pharmaceutical preparations.

### Human growth hormone

22 kDa hGH, also known as somatotropin, is a single chain polypeptide consisting of total 191 amino acids [22,23]. It is synthesized and secreted by cells known as somatotrophs in the anterior pituitary. In the lifetime of an individual, the highest quantities of growth hormone are produced during puberty in the anterior lobe or glandular portion of the pituitary [24-28]. hGH is synthesized as a precursor and released into the blood following post-translational modifications. The hormone is used in the treatment of certain forms of dwarfism caused by hGH deficiencies, in obesity therapy, and in wound and burn treatment. Clinical trials indicate that hGH helps diminish the devastating effects of the hypermetabolic response to burn injury especially among children. These are the reasons why the demand for this hormone is very high [29-33].

Production of recombinant hGH requires optimization of vectors used to express the protein in those heterologous hosts. A series of methods have been designed [8,34-39]. A generic expression vector features promoter sequences facilitating transcription and translation as well as sequences ensuring the stability of the synthesized protein. The vector described in this study contains strong promoters that enable efficient synthesis and accumulation of 30% or more of the targeted recombinant proteins among the total cellular proteins. In this study, we have reported the cloning, expression, refolding, and purification of hGH.

## Results and discussion

### The Ub tag was used to obtain a mature form of hGH

**Figure 1:**
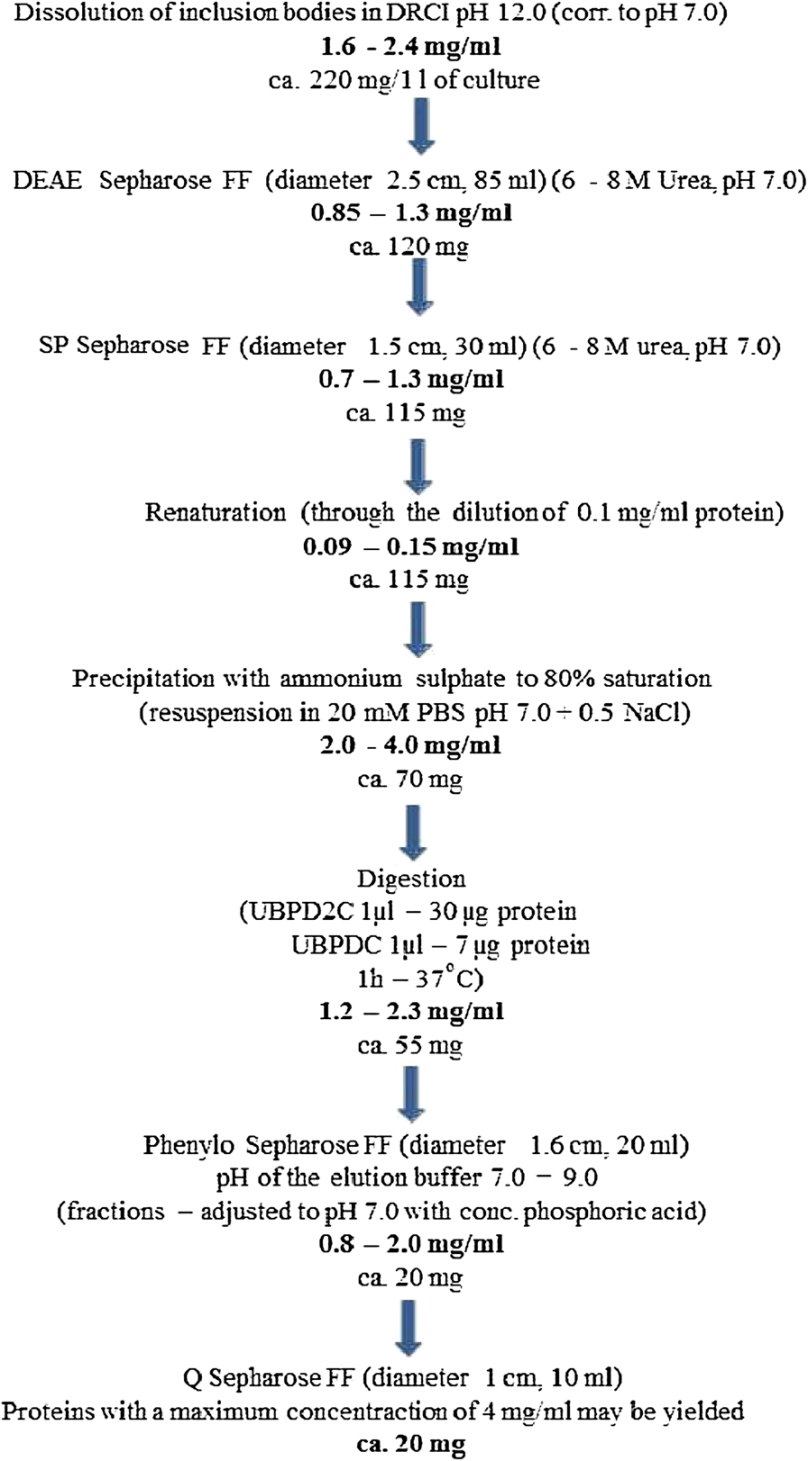
**Schematic showing the steps involved in the purification of the growth hormone.** The values obtained for selected parameters of the process as well as the protein yields are indicated.

Ub, the best-known short protein, was used as the tag in this study. The use of Ub to enhance the levels of protein expression has been reported previously [4,13,14,40-43]. In the present study, efficient expression of the Ub::hGH fusion gene in *E. coli* strains was achieved, and the UBPD2C analog of the UBP1 protease was used to cleave the Ub moiety from the N-terminus of the hGH. The hybrid polypeptide gene was cloned into several expression vectors such as pIGAL1, pIGDM1, pIGRKAN, and pIGMS31KAN. The resulting plasmids pIGDMKUH, pIGALUH, pIGALUHM, pIGRKKhGH, and pIGMS31PRH were used to transform *E. coli* strains DH5α and NM522. The *E. coli* DH5α cells were found to be most suitable with regard to the expression level of this fusion gene and the protein purification process. We used several vectors to determine the most suitable one, and the differences between them have been described in the Materials and methods section. The above-mentioned expression vectors with constitutive promoters *pms* or *P1, P2* and terminators T1, T2 and *pms* (in appropriate combinations) were used. All of the constructed *E. coli* strains carried one of the following plasmids: pIGDMKUH, pIGALUH, pIGALUHM, pIGRKKhGH, or pIGMS31PRH, and were tested according to the procedure briefly presented in Figure 1.

**Figure 2:**
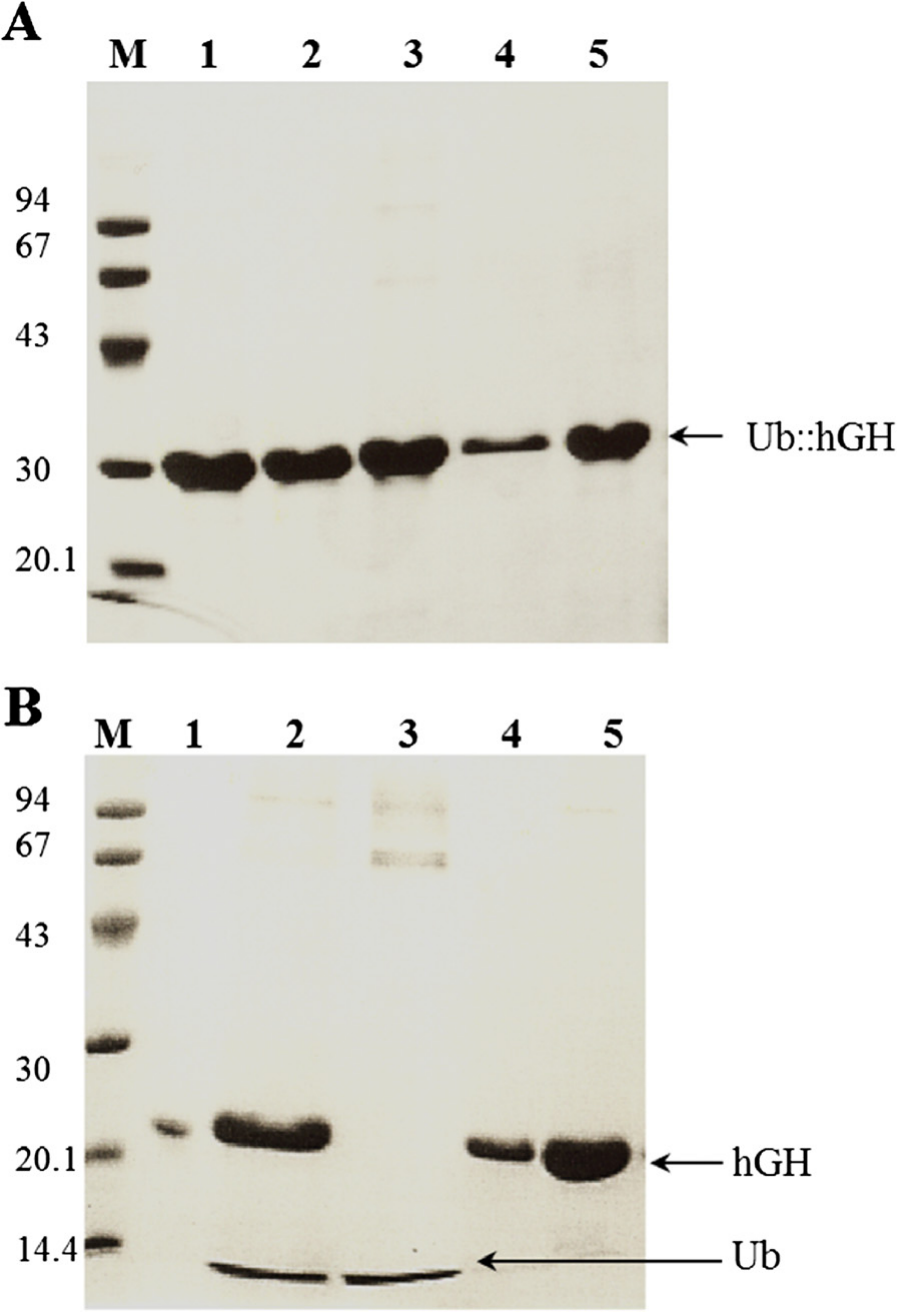
**SDS-PAGE of the growth hormone samples from successive purification stages. (A)** M, low molecular weight (LMW) protein marker (94, 67, 43, 30, 20.1, and 14.4 kDa); 1, the protein dissolved in DRCI buffer; 2, peak I obtained after purification on DEAE Sepharose FF column; 3, peak I obtained after purification on SP Sepharose FF column; 4, protein after renaturation; 5, sample after ammonium sulfate precipitation. **(B)** M, low molecular weight (LMW) protein marker (94, 67, 43, 30, 20.1, and 14.4 kDa); 1, growth hormone European standard; 2, Phenyl Sepharose FF column entry; 3, fraction I obtained after purification on Phenyl Sepharose FF column; 4, fraction II obtained after purification on Phenyl Sepharose FF column; 5, fraction III obtained after purification on Phenyl Sepharose FF column.

**Figure 3:**
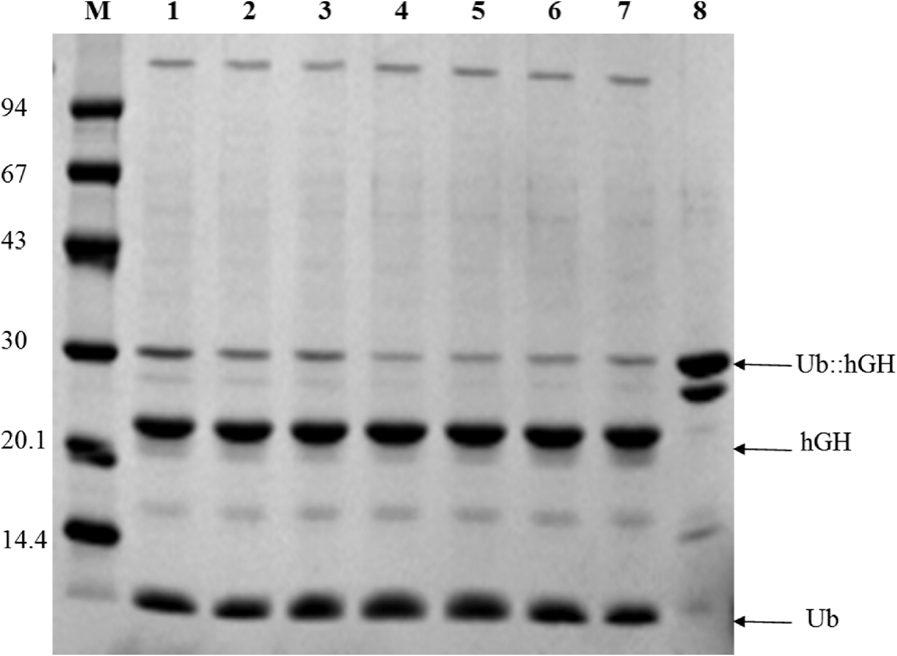
**SDS-PAGE of Ub::hGH fusion protein cleaved by UBPD2C protease: M, low molecular weight (LMW) protein marker (94, 67, 43, 30, 20.1, and 14.4 kDa); 1, 10-min digestion; 2, 15-min digestion; 3 and 7, 30-min digestion; 4, 40-min digestion; 5, 50-min digestion; 6, 60-min digestion; 8, standard Ub::hGH.**

The scheme presented in Figure 1 summarizes the workflow after harvest of the inclusion bodies: purification of Ub::hGH by chromatography on SP Sepharose FF column, renaturation, protein precipitation and cleavage of Ub from Ub::hGH fusion protein. Purification of hGH was performed on Phenyl Sepharose FF column and then the protein was concentrated on Q Sepharose FF column. The purity of the preparations at each stage of purification was monitored via SDS-PAGE (Figures 2 and 3).

### Ub::hGH cleavage by UBPD2C-the UBP1 analog

The time course of Ub::hGH fusion protein digestion by the UBP1 protease analog was analyzed. Aliquots of the reaction mixture were collected at different time points and resolved by SDS-PAGE (Figure 3). It can be clearly seen from the figure that most of the Ub:hGH was converted to hGH already after 10 min. The reaction proceeds up to 40 min. From that point the intensity of the Ub::hGH band does not change indicating termination of the cleavage.

**Figure 4:**
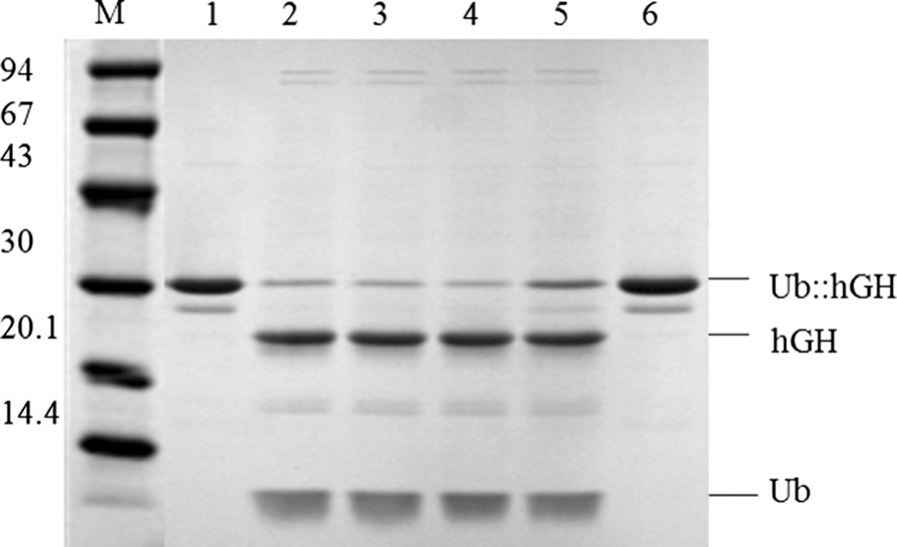
**SDS-PAGE analysis of the UBPD2C activity after incubation for 22 h at temperatures of 4°C, 20°C, 25°C and 37°C.** 1, 6 Ub::hGH undigested; 2, incubated at 4°C; 3, incubated at 20°C; 4, incubated at 25°C; 5, incubated at 37°C; M, low molecular weight standard (kDa).

The stability of the UBPD2C enzyme prepared in an earlier study [19] was further investigated. The UBP1 protease analog was incubated for 22 h at temperatures of 4°C, 20°C, 25°C and 37°C, respectively. Subsequently, each of these test preparations was used for digesting the Ub::hGH fusion protein under the conditions described in the Materials and methods section. Aliquots of the samples collected at different time points were analyzed by SDS-PAGE. As shown in Figure 4, the protease was stable at all the different temperatures tested (Figure 4).

**Figure 5:**
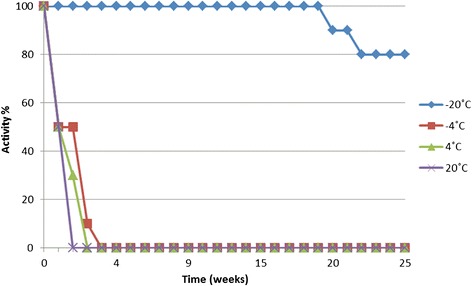
**Stability of UBPD2C protease.** Stability of UBP1 protease measured every week after incubation of this enzyme at +20°C, +4°C, -4°C or -20°C. The first measurement of UBP1 protease activity incubated at +20°C followed after 3 days.

Furthermore, the stability of the purified UBP1 analog protease was also tested by storing aliquots of the samples at 20°C, 4°C, -4°C and -20°C for 25 weeks. Subsequently, standard digestion procedure using the Ub::hGH purified substrate was carried out and the extent of cleavage was assessed by SDS-PAGE, followed by densitometric quantification of the reaction products (GelScan v. 1.45). The results of this analysis, shown in Figure 5, clearly demonstrated that UBPD2C enzyme can be stored for 25 weeks at -20°C. If stored at 4°C it is completely inactivated after 2 weeks and at -4°C after 3 weeks. Incubation of the protease at 20°C for three days allowed to retain 50% of its activity. After 1 week in this temperature it became completely inactivated. Protease stored at -70°C remained active for 25 weeks (data not shown). The enzyme is capable working in a wide range of pH (from pH 10.0 to pH 6.0) and temperature (from 15°C to 50°C) (data not shown).

Final preparations of the hGH purified from the five tested *E. coli* strains were analyzed by high-performance liquid chromatography (HPLC) using the method described in the European Pharmacopoeia (5^th^ Edition, 2005) [44]. Accordingly, *E. coli* DH5α cells carrying the pIGDMKUH plasmid were found to be the best construct for obtaining hGH. The hGH preparation obtained from this strain and purified according to the method described earlier exhibited approximately 92.2% purity, thus fulfilling the specifications/conditions required by United States Pharmacopeial Convention [45] and European Pharmacopoeia (7^th^ Edition, 2010) [44] (Table 1). Accordingly, in order to monitor the size of molecules present in the Ub::hGH UBPD2C digest and hGH after Phenlyl Sepharose FF Column, size exclusion chromatography (SEC) was performed (data not shown, please refer to the Figure 1 in the manuscript, showing the steps involved in the purification of the growth hormone). The protein purity analysis allowed to observe a significant improvement in hGH purity by removal of high and low molecular weight impurities present in the digest.

**Table 1:** **Results of culturing, isolation of inclusion bodies, and chromatographic purification of hGH from** ***E. coli*** **DH5α cells carrying the pIGDMKUH plasmid**

**Phase – Ub/hGH**		**Volume (ml)**	**Protein**		
			**(mg/ml)**		**(mg) (%)**
Dissolved inclusion bodies		200	2.2		443.3 (100)
DEAE	Column entry	200	2.2		443.3 (100)
Sepharose					
Fast Flow	Fraction	184	1.32		242.8 (54.7)
Column					
SP	Column entry	184	1.32		242.8 (54.7)
Sepharose					
Fast Flow	Fraction	184	1.25		230 (51.8)
Column					
Protein after renaturation		1680	0.14		230 (51.8)
Protein after ammonium sulfate precipitation to 80% saturation		50	2.85		142.5 (32.1)
**Phase – hGH**		**Volume (ml)**	**Protein**		**HPLC purity (%)**
			**(mg/ml)**	**(mg) (%)**	
Phenyl	Column entry	64	1.73	110.7 (24.9)	50
Sepharose					
Fast Flow	Fractions	50	0.85	42.4 (9.56)	91.8
Column					
Q	Column load	50	0.85	42.4 (9.56)	91.8
Sepharose					
Fast Flow	Fractions	10	4.02	40.2 (9.06)	92.2
Column					

### Analysis of the stability of various hGH expression constructs in *E. coli* DH5α cultures grown in an antibiotic-free LB medium

Stability experiments were conducted according to the above-described method using the *E. coli* DH5α host strain with various expression vectors: pIGMS31PRH, pIGRKKhGH, pIGDMKUH, pIGALUH, pIGALUHM.

**Figure 6:**
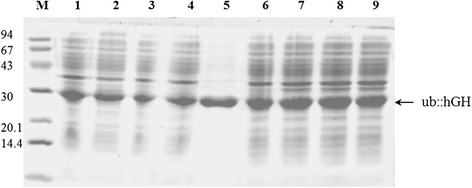
**SDS-PAGE assessment of the stability of the pIGDMKUH plasmid in** ***E. coli*** **DH5α cells.** Every passage was cultured for 18 h. M, low molecular weight standard (94, 67, 43, 30, 20.1, and 14.4 kDa); 1, 1^st^ passage of culture with antibiotic pressure; 2, 2^nd^ passage of culture with antibiotic pressure; 3, 3^rd^ passage of culture with antibiotic pressure; 4, 4^th^ passage of culture with antibiotic pressure; 5, purified Ub::hGH as control; 6, 1^st^ passage of culture without antibiotic pressure; 7, 2^nd^ passage of culture without antibiotic pressure; 8, 3^rd^ passage of culture without antibiotic pressure; 9, 4^th^ passage of culture without antibiotic pressure.

SDS-PAGE and phase contrast microscopy revealed that the most stable plasmids used for the transformation of *E. coli* DH5α cells was pIGDMKUH. The plasmid was found to remain stable through 80 generations (four passages) in antibiotic-free cultures (Figure 6). This finding is important for the use of the protein in the pharmaceutical industry.

Production of hGH through fermentation using organisms transformed by recombinant hGH expression constructs requires optimization of vectors used to express the proteins of interest in those heterologous hosts. A generic expression vector features promoter sequences facilitating transcription and translation as well as sequences ensuring the stability of the synthesized protein. The vectors described in this study contained strong promoters that could direct efficient synthesis and accumulation of 30% or more of recombinant proteins among the total cellular proteins. Each construct was different with respect to the kind of promoter used or its orientation or transcription terminator or resistance to antibiotics. These were the main variations leading to selection of one final vector construct enabling most efficient recombinant hGH expression.

In many cases the overall yield of biologically active protein is very low, and the methods of purification hGH are often not efficient or time-consuming. An alternative approach that has been developed involve complete solubilization and purification of rhGH produced in *E. coli*. [34]. Kim et al described obtaining of hGH and His-hGH expression by inducing IPTG and purifying them on the Ni-NTA column. The other work [46] described seven N-terminal fusion partners His6, Trx, GST, b’ and a’ domain of PDIb’ a’, NusA, PDI for soluble overexpression of hGH in *E. coli*. To cleave the tag proteins from fused hGH, the TEVrs protease was used. The tobacco etch virus recognition site ENLYFQ/G was placed at the N-terminus of hGH. In this case, only the DNA sequence was changed rather than amino acids. We tried to obtain hGH with a native growth hormone sequence for medical/pharmaceutical application.

In addition to the above-mentioned analyses, HPLC purification, sequencing of the N-terminus (15 amino acid residues; performed at Biocenter Kraków), and mass spectrometry of the purified preparation were performed to confirm that the protein is hGH.

### Biological activity of rhGH

To determine the biological activity of the obtained rhGH, the protein was allowed to stimulate mitosis, and then tetrazolium salt (MTT) was added to determine the number of live cells. In this method, active mitochondrial dehydrogenases in the live cells changed the yellow color of the MTT to a purple product of formazan with a concentration proportional to the number of live cells.

**Figure 7:**
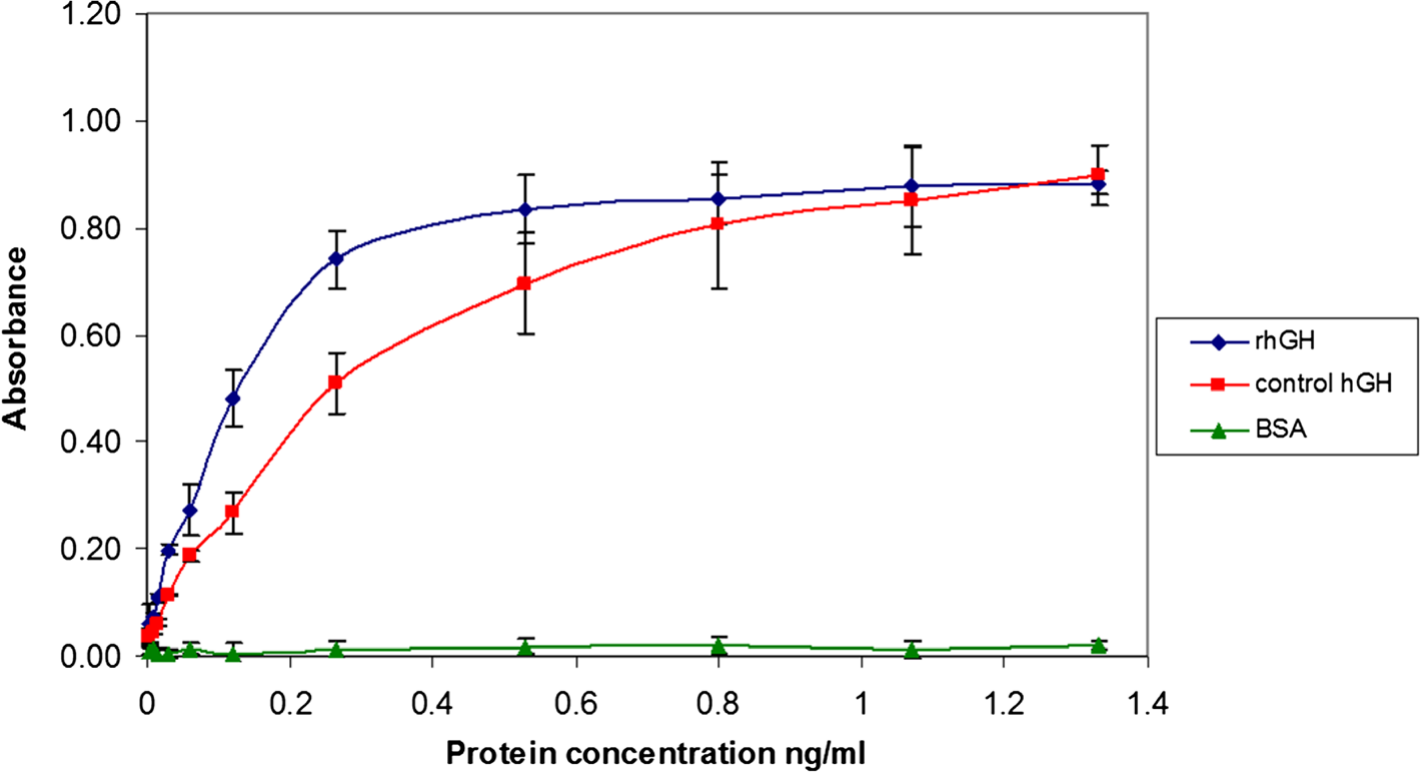
**Results of the measured biological activity of the prepared rhGH obtained with the use of KC Junior program in ELISA μQuant Bio-Tek Instruments.** (■) control hGH (somatotropin) curve; (♦) curve for absorbance vs. rhGH protein concentration in the preparation and (▲) BSA at the indicated concentration.

This method allowed the examination of the biological activity of the rhGH preparation, and confirmed that proper tertiary structure of the protein had been obtained. The calculated biological activity of the preparation was 4.4 IU/mg and that of the somatotropin standard was 2.0 IU/mg. The rhGH protein purified under our conditions retained its biological activity at the comparable level to that of control hGH. In contrast, no growth stimulation was observed with BSA, which was used as a negative control (Figure 7).

Once satisfactory yield of purified hGH was obtained from 1 l of bacterial culture, the process was further scaled-up and the growth hormone was produced on a large scale (data not shown). Usage of 150 liter fermentor enabled to achieve the production capacity of 250 g per year.

## Conclusions

The purpose of this study was to develop an efficient method for obtaining recombinant hGH from *E. coli*. We showed that the strains of *E. coli,* transformed with expression plasmids carrying the Ub-growth hormone gene, yielded satisfactory expression of the Ub-growth hormone protein in LB medium. In each case, most of the Ub-growth hormone fusion protein was produced in the inclusion bodies. The efficiency of protein expression was found to be sufficiently high, enabling the developed strains of *E. coli* to be used for industrial-scale production of hGH (220 mg of inclusion bodies/1 l of culture). This method could facilitate the production of relatively large quantities of the protein with appropriate quality (purity: 92.2%) and activity (4.4 IU/mg while the standard reached 2.0 IU/mg) and application in pharmaceutical industry.

Furthermore, another purpose of this study was to establish a protocol for growth hormone purification, in particular, the renaturation conditions. Accordingly, it was found that the UBP1 protease analog is a flexible tool that may be successfully used in experimental research as well as in the manufacture of recombinant proteins.

To summarize, the use of the above-mentioned system (expression of the final substrate in the form of Ub::hGH fusion protein and the use of UBP1 analog protease for deubiquitination), exploiting a highly specific and stable protease allows retrieving the required protein with high yield. 20 mg of the purified hGH was obtained from 1 l of bacterial culture. These properties make it suitable for a large-scale production in the pharmaceutical industry. Moreover, it must be noted that the *E. coli* DH5α strain with pIGDMKUH plasmid was the most efficient construct, which remained stable and facilitated bacterial growth in the absence of any antibiotic.

## Materials and methods

### Primers, plasmids, and bacterial strains

The UBPD2C protease gene was obtained as described previously [19,20], [47]. The Ub::hGH fusion gene was cloned into plasmid pIGCMST derivative [48] using restriction enzymes, *Nde*I and *Sal*I. The recombinant plasmid was named pIGCMUHGH and was deposited in GenBank (Accession No. HQ845201) [49].

The pIGCMUHGH plasmid was used as a template for PCR. The hGHubF and hGHubF primers were used (Table 2) to amplify the Ub::hGH sequence. The amplified 450-bp DNA fragment was purified using a DNA gel extraction kit (A&A Biotechnology, Gdansk, Poland), and was digested with *Nde*I and *Sal*I and subcloned into pIGMS31KAN, pIGRKKAN, pIGDMKUH, pIGALUH, and pIGALUHM expression vectors. Each of the obtained plasmids was sequenced to confirm the identity of the DNA sequence. Subsequently, these plasmids were transformed into *E. coli* strain NM522. The genotypes of the bacterial strains used in this study are listed in Table 3. The strains were grown in Luria-Bertani (LB) medium as described previously [50].

**Table 2:** **Nucleotide sequences of primers used for PCR and construction of Ub::hGH fusion gene**

**Name of primer**	**DNA sequence (restriction sites underlined)**	***T***_**m**_ **(°C)**	**Restriction site**
hGHubF	GGGG*CCGCGG*TTCCCAACCATTCCCTTAAGTAGGC	57	R.*SacII*
hGHubR	GGGG*GTCGAC*TTAGAAGCCACAGCTGCCCTCC	58	R.*Sal*I

**Table 3:** **Bacterial strains used in this study**

**Strains**	**Genotype**	**Source**
*E. coli* NM522	F‾ proA + B + lacIq Δ(*lacZ*)M15/Δ(*lac-proAB*) glnV thi-1 Δ(*hsdS-mcrB*)5	Stratagene
*E. coli* DH5α	F‾, Φ80dlacZΔM15Δ(*lacZYA-argF*)U169, deoR, recA1, endA1, hsdR17(rK- mK+), supE44, λ–, thi-1, gyrA96, relA1	Novagen

### Construction of expression vectors

#### *pms* promoter and deo P1 P2 promoter

The *pms* promoter was isolated from a plasmid present in a clinical strain of *Klebsiella pneumonia* (GenBank Accession No. AY543071) [49,50]. It is a strong, constitutive promoter (Patent PL 213561). The sequence of the deo P1P2 promoter/operator region is known and the entire deo P1P2 promoter region is approximately 760 bp long, with the two promoters being separated by a distance of about 600 bp. Expression of a gene product under deo P2-driven transcription is very low in the presence of glucose and very high in the presence of other energy producing sources. Initiations from P1 are negatively controlled by the deoR repressor, the inducer being deoxyribose-5-phosphate (deoxyribose-5-P). Initiations from P2 depend on the cyclic AMP/cyclic AMP receptor protein complex (cAMP/CRP) and are negatively controlled by the cytR repressor, the inducer being cytidine [51].

#### pIGMS31KAN plasmid

**Figure 8:**
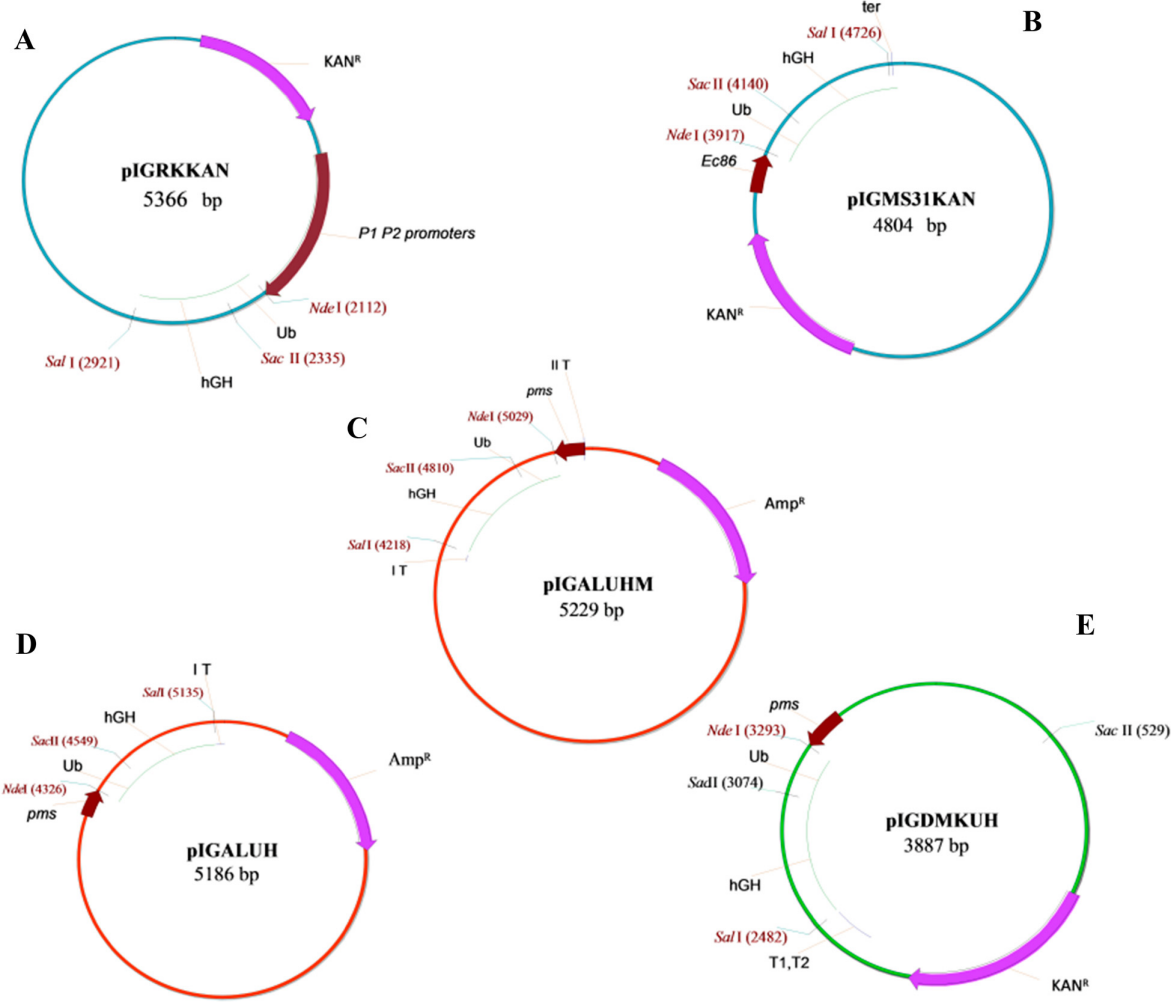
**Schematic representation of the expression vectors A-pIGRKKAN, B-pIGMS31KAN, C-pIGALUHM, D-pIGALUH and E-pIGDMKUH.** hGH was expressed as a fusion protein with the Ub tag. The restriction enzyme sites used to clone the Ub::hGH gene are indicated.

The promoter sequence of retron Ec86 and its following transcription terminator sequence [52] were inserted into pIGMS31KAN plasmid. The resulting pIGMS31PR plasmid was used to clone the Ub::hGH fusion gene (Figure 8).

#### pIGRKKAN plasmid

P1 and P2 deo operon of *E. coli* strain K-12 promoters, and the gene encoding the fusion protein composed of synthetic Ub and the gene growth hormone in R.*Nde*I and R.*Sal*I restriction sites were cloned into pIGRKKAN plasmid, which was isolated from *K. pneumonia* strain 287-w. The restriction map of the recombinant plasmid is presented in Figure 8.

#### pIGDMKUH plasmid

The pIGDMKUH plasmid was derived from pIGDM1 plasmid (GenBank Accession No. AF014880) [49]. The length of the pIGDMKUH plasmid was 3887 bp and contained the *pms* promoter and T1, T2 transcription terminators [53]. The synthetic Ub::hGH fusion gene was cloned into R.*Nde*I and R.*Sal*I sites (Figure 8).

#### pIGALUH and pIGALUHM plasmids

The pIGALUH and pIGALUHM plasmids were derived from pIGAL1 plasmid (GenBank Accession No. AY424310). The synthetic Ub::hGH fusion genes were cloned into the pIGALUH and pIGALUHM plasmids. The pIGALUH plasmid contained the ampicillin resistance gene, *pms* promoter, and terminator sequences, while the pIGALUHM plasmid contained the ampicillin resistance gene, *pms* promoter in reverse orientation, as well as an additional transcription terminator located upstream of the *pms* promoter (Figure 8).

### Creating bacterial glycerol stocks for long-term storage of plasmids

The method of preparing bacterial stocks is critical for maintaining efficient expression and plasmid stability in host cells. One bacterial colony was inoculated into 3 ml of LB medium with an appropriate antibiotic. The culture was incubated under shaking for 4 h at 37°C, and then transferred into 20 ml of LB medium with antibiotic and again incubated under shaking at 37°C until the optical density measured at 600 nm (OD_600_) reached ≈ 1. The culture was subsequently aliquoted into sterile Eppendorf tubes (0.5 ml/tube) and 0.5 ml of 40% glycerol was added to each tube. The bacterial stocks were stored at −70°C. Later, 0.5 ml of the stock was added to 1 l of LB medium, and 200 ml of the LB medium supplemented with 200 μl of ampicillin (100 μg/ml) or 200 μl of kanamycin (50 μg/ml) and 100 μl of the inoculate was added to a 500-ml flask. The culture was incubated in a rotary shaker at 160 rpm and 37°C for 18 h, and then centrifuged at 6000 rpm for 5 min at 4°C. The obtained bacterial cell pellet was processed as described in the subsequent section.

### Purification of Ub::hGH and cleavage of the fusion protein by deubiquitinating UBPD2C

#### Isolation of inclusion bodies

Wet cell biomass of 5–7 g, obtained from 1 l of culture, was suspended in 100 ml of Buffer C (50 mM Tris HCl, 0.5 M NaCl, and 5 mM β-mercaptoethanol; pH 7.5), and lysozyme was added to a final concentration of 0.43 mg/ml. The suspension was incubated for 35 min at 20°C. Then, Triton X-100 was added to a final concentration of 1% and the mixture was sonicated. Subsequently, phenylmethylsulfonyl fluoride (PMSF) was added to a final concentration of 1 mM and then glycerol followed in a quantity corresponding to one-third of the mass of the cell suspension. The mixture was centrifuged at 8000 rpm for 20 min at 20°C. The precipitate containing inclusion bodies was suspended in 100 ml of CT buffer (50 mM Tris HCl, 0.5 M NaCl, 5 mM β-mercaptoethanol, and 1% Triton X-100; pH 7.5) and sonicated for 10 min on ice, as previously described [50]. After centrifugation at 8000 rpm for 20 min, the precipitate was suspended in 100 ml of phosphate buffered saline (PBS) with 1% Triton X-100 and sonicated [50]. The precipitate was centrifuged again and suspended in 100 ml of PBS with 2 M urea, sonicated as reported [50], and centrifuged for another time to obtain the inclusion bodies. The yield of inclusion bodies was between 0.7 and 1.2 g/l of culture.

The inclusion bodies were dissolved in DRCI buffer (6–8 M urea, 50 mM phosphate buffer, and 5 mM β-mercaptoethanol; pH 12). The solution was cleared by centrifugation, its pH was adjusted to 7.0 with concentrated phosphoric acid, and was subsequently loaded onto DEAE Sepharose fast flow (FF) column (approximately 100 ml; GE Healthcare Life Sciences) equilibrated with 6–8 M urea and 20 mM phosphate buffer at pH 7.0. The flow through material, containing the Ub::hGH protein, was collected and the fusion protein was loaded onto SP Sepharose FF column.

#### SP Sepharose FF column

The pooled Ub::hGH peak fractions from the DEAE Sepharose FF column were directly loaded onto a 30-ml column filled with SP Sepharose FF (Amersham Pharmacia Biotech AB) equilibrated with 6–8 M urea and 20 mM phosphate buffer at pH 7.0. The flow through material, containing the Ub::hGH protein, was collected and renatured. Separation on this column was not necessary if the Ub::hGH protein yield from the DEAE column was about 70%.

#### Renaturation

Ub::hGH in fractions from the SP Sepharose FF column was renatured by approximately 10-fold dilution in BR buffer (20 mM phosphate buffer pH 7.0, 50 mM NaCl) to a concentration of 0.09–0.15 mg/ml protein and then incubated for 1 h at room temperature.

#### Ammonium sulfate precipitation

After renaturation, the fusion protein was precipitated by adding ammonium sulfate to 80% saturation at 4°C under constant stirring. The samples were centrifuged at 12000 rpm for 15 min at 4°C. The pellet was suspended in 1/40th of the pre-centrifugation sample volume (approximately 50 ml) of 20 mM phosphate buffer (pH 7.5) and 0.5 M NaCl, and then centrifuged at 12000 rpm for 15 min at 4°C.

#### Cleavage of Ub::hGH fusion protein using UBPDC, UBPD2C protease

The supernatant (protein in 20 mM phosphate buffer (pH 7.5) + 0.5 M NaCl) was incubated with appropriate DUB preparations to cleave the Ub moiety from the growth hormone polypeptide. The reaction was performed at 37°C for 1 h (1 μl of UBPD2C 60 μg of protein; 1 μl of UBPDC – 14 μg of protein). Then, the sample was centrifuged at 12000 rpm for 15 min at 4°C. The supernatant was fractionated by chromatography on Phenyl Sepharose FF resin. The purification process of UBPD2C and UBPDC proteases has been described elsewhere [19,47].

#### Phenyl Sepharose FF column

Approximately 20 ml of Phenyl Sepharose FF column (GE Healthcare Life Sciences) was equilibrated with 0.5 M NaCl and 20 mM phosphate buffer (pH 7.0). The hGH protein was eluted with a 3 mM phosphate buffer at pH 7.0–9.0. The fractions containing the growth hormone were collected, and the protein solution was adjusted to pH 7.0 using concentrated phosphoric acid and stored at 4°C.

#### Q Sepharose FF column

The growth hormone fractions from Phenyl Sepharose FF column were concentrated on a 10-ml Q Sepharose FF column (Amersham Pharmacia Biotech AB). The carrier was equilibrated with 20 mM phosphate buffer at pH 7.5, transferred to the column, and eluted with a linear gradient of 0.5 M NaCl in 20 mM phosphate buffer (growth hormone containing fractions were eluted at 0.25 M NaCl).

### Storage

The purified growth hormone was transferred into 3.1 mM Na_2_HPO_4_ (pH 7.0; by hydrophobic interaction chromatography, carrier: Phenyl Sepharose FF, isocratic distribution) for storage at 4°C.

### Verification of plasmid stability in cultures grown in LB medium without antibiotic

One stock was inoculated into 50 ml of antibiotic-free LB medium. The culture was incubated under shaking at 37°C until an OD_600_ of ~1 was reached. Subsequently, 1 ml of the culture was used for successive passages. Four passages were carried out for each culture for 18 h, and 1-ml samples were collected to obtain bacterial lysates. The Ub::hGH gene expression was verified in the host cells. The experiment was repeated four times independent of the transformation phase, and the accuracy of the results was confirmed.

### Densitometric analysis using Scangel v. 1.45 program

Quantitative analyses of the proteins Ub::hGH, Ub, and hGH were performed by staining with 0.05% Coomassie Brilliant Blue R250 and obtaining electropherograms using the software package Scangel v. 1.45 program (Kucharczyk T.E., Warsaw, Poland). For destaining, the gels were incubated in the same solution without the dye.

### Biological activity assay method

The hGH biological activity assay in the Nb-2 rat cell line was carried out as described previously [51,54-56]. Nb-2 is a prolactin-related cell line sensitive to hGH (somatotropin; European Collection of Cell Cultures (ECACC)). The Nb-2 cells were prepared for the hGH assay as described by Tanaka et al. [57]. After a 24-h incubation period, the cells were suspended at a concentration of 1 × 10^5^ cell/ml in RPMI-1640 medium containing 10% horse serum, glutamine, antibiotic, and 2-mercaptoethanol. Subsequently, 100 μl of the suspension were added to each well of a 96-well cell culture plate, flat bottom (Nunc).

#### hGH standard solution

Somatotropin (2.0 IU/mg protein specific activity (SIGMA S-4776)) was used as the standard for hGH biological activity assay. The somatotropin solution was distributed onto a plate with Nb-2 cells so that its concentrations on the plate were 0.004, 0.008, 0.015, 0.03, 0.06, 0.12, 0.265, 0.53, 0.8, 1.07, and 1.33 ng/ml. Six replicates were assayed for each somatotropin concentration.

#### Preparation of dilutions of the test hGH preparation

The growth hormone protein concentration in the test preparation was assayed at a wavelength of 280 nm, considering the amount of tyrosine, tryptophan, and cysteine in the hGH. Solutions of the growth hormone preparation were distributed onto a plate with Nb-2 cells in six replicates. The scheme of solutions distribution and their concentrations were similar to those employed for somatotropin, the commercial marker of hGH.

#### Absorbance reading

The plates were incubated for 72 h (37°C, 5% CO_2_). Subsequently, 15 μl of MTT solution (5 mg MTT/1 ml of PBS) were added to each well, and the plates were incubated for 3 h at 37°C. Then, 60 μl of lysis buffer (10% Triton X-100, 0.2 N HCl, and 20% isopropanol) were added to each well and gently stirred. After that, 50 μl of DMSO were added and the plates were incubated for 0.5 h at 37°C. The absorbance was read with an ELISA plate reader (μQuant Bio-Tek Instruments) at two wavelengths: 585 and 655 nm. The somatotropin standard curves and hGH activity were determined using the KC Junior software program.

## Abbreviations

aa: Amino-acid residue
bp: Base pair(s)
DUB: Deubiquitinating protease
IPTG: Isopropyl-β-D-galactoside
hGH: Human growth hormone
kDa: Kilodalton
LB: Luria-Bertani
PCR: Polymerase chain reaction
IU: International unit
UB: Ubiquitin
UBP: Ubiquitin-specific processing protease
SDS: Sodium dodecyl sulfate
SDS-PAGE: Sodium dodecyl sulfate-polyacrylamide gel electrophoresis
